# Supplementary material for: Hot spot prediction in protein-protein interactions by an ensemble system
Source: BMC Syst Biol. 2018 Dec 31;12(Suppl 9):132. doi: 10.1186/s12918-018-0665-8 (PMC6311905; doi:10.1186/s12918-018-0665-8)
Supplement: Supplementary file 2 — All relevant MATLAB code. A simple Matlab implement of our predictor. (ZIP 19,742 kb) [file 12918_2018_665_MOESM2_ESM.zip › Additional file 2/Read ASA/ASA Property/BID/3SAKA.txt]

 NetSurfP - Protein Surface Accessibility and Secondary Structure Predictions

  

|  |  |  |
| --- | --- | --- |
|  |  | NetSurfP - Protein Surface Accessibility and Secondary Structure Predictions  Technical University of Denmark |

  


---

```
# For publication of results, please cite:
# A generic method for assignment of reliability scores applied to solvent accessibility predictions.
# Bent Petersen, Thomas Nordahl Petersen, Pernille Andersen, Morten Nielsen and Claus Lundegaard
# BMC Structural Biology 2009, 9:51 doi:10.1186/1472-6807-9-51
#
# Column 1: Class assignment - B for buried or E for Exposed - Threshold: 25% exposure, but not based on RSA
# Column 2: Amino acid
# Column 3: Sequence name
# Column 4: Amino acid number
# Column 5: Relative Surface Accessibility - RSA
# Column 6: Absolute Surface Accessibility
# Column 7: Z-fit score for RSA prediction
# Column 8: Probability for Alpha-Helix
# Column 9: Probability for Beta-strand
# Column 10: Probability for Coil
E K  3SAKA                  1    0.904 185.871   1.050   0.003   0.003   0.994
E K  3SAKA                  2    0.579 119.162  -0.380   0.019   0.141   0.840
E K  3SAKA                  3    0.591 121.651   0.176   0.019   0.141   0.840
E P  3SAKA                  4    0.339  48.118  -1.573   0.018   0.088   0.893
E L  3SAKA                  5    0.350  64.140  -1.008   0.052   0.084   0.864
E D  3SAKA                  6    0.680  97.930  -0.132   0.052   0.084   0.864
E G  3SAKA                  7    0.568  44.670  -1.046   0.056   0.142   0.802
E E  3SAKA                  8    0.503  87.874  -0.084   0.022   0.359   0.619
E Y  3SAKA                  9    0.303  64.751   0.336   0.021   0.756   0.223
B F  3SAKA                 10    0.083  16.658   0.696   0.018   0.846   0.136
B T  3SAKA                 11    0.244  33.843   0.309   0.011   0.918   0.071
B L  3SAKA                 12    0.069  12.725   0.091   0.011   0.918   0.071
E Q  3SAKA                 13    0.348  62.171   0.853   0.011   0.918   0.071
B I  3SAKA                 14    0.071  13.191  -0.777   0.018   0.846   0.136
E R  3SAKA                 15    0.422  96.661   0.419   0.074   0.484   0.442
B G  3SAKA                 16    0.229  18.014  -1.136   0.125   0.227   0.648
E R  3SAKA                 17    0.421  96.501   0.566   0.428   0.171   0.402
E E  3SAKA                 18    0.532  92.975  -0.129   0.605   0.105   0.290
B R  3SAKA                 19    0.190  43.556   0.315   0.879   0.010   0.111
B F  3SAKA                 20    0.114  22.820   0.504   0.938   0.007   0.055
E E  3SAKA                 21    0.486  84.834   1.221   0.938   0.007   0.055
B M  3SAKA                 22    0.107  21.471  -0.077   0.970   0.001   0.030
B F  3SAKA                 23    0.044   8.851   0.140   0.970   0.001   0.030
E R  3SAKA                 24    0.390  89.356   1.122   0.970   0.001   0.030
E E  3SAKA                 25    0.477  83.349   1.180   0.923   0.002   0.076
B L  3SAKA                 26    0.046   8.441   0.376   0.923   0.002   0.076
B N  3SAKA                 27    0.289  42.266  -0.136   0.858   0.002   0.139
E E  3SAKA                 28    0.537  93.779   0.697   0.858   0.002   0.139
B A  3SAKA                 29    0.164  18.062  -1.329   0.879   0.010   0.111
B L  3SAKA                 30    0.131  23.931   0.749   0.717   0.014   0.269
E E  3SAKA                 31    0.537  93.814   0.592   0.561   0.047   0.393
B L  3SAKA                 32    0.134  24.609   0.330   0.455   0.046   0.498
E K  3SAKA                 33    0.495 101.904   0.385   0.455   0.046   0.498
E D  3SAKA                 34    0.521  75.033  -0.064   0.561   0.047   0.393
B A  3SAKA                 35    0.285  31.352  -0.811   0.622   0.015   0.363
E Q  3SAKA                 36    0.496  88.532  -0.460   0.622   0.015   0.363
E A  3SAKA                 37    0.367  40.399  -0.836   0.522   0.016   0.462
E G  3SAKA                 38    0.488  38.429  -1.019   0.430   0.016   0.555
E K  3SAKA                 39    0.641 131.874  -0.449   0.257   0.016   0.727
E E  3SAKA                 40    0.605 105.659  -0.532   0.115   0.016   0.868
E P  3SAKA                 41    0.653  92.703  -1.966   0.058   0.017   0.925
E G  3SAKA                 42    0.939  73.876  -1.623   0.003   0.003   0.994

Explain the output.  Go back.
```

---
